# Propionic acid induces dendritic spine loss by MAPK/ERK signaling and dysregulation of autophagic flux

**DOI:** 10.1186/s13041-020-00626-0

**Published:** 2020-06-02

**Authors:** Hyosun Choi, In Sik Kim, Ji Young Mun

**Affiliations:** 1 BK21 Plus Program, Department of Senior Healthcare, Graduate School, Eulji University, Daejeon, South Korea; 2 Neural Circuit Research Group, Korea Brain Research Institute, Daegu, 41068 Republic of Korea; 3 Department of Biomedical Laboratory Science, Eulji University School of Medicine, Daejeon, South Korea

**Keywords:** Propionic acid, Short-chain fatty acid, Autophagy, MAPK/ERK signaling, Spine density

## Abstract

Propionic acid (PPA) is a short-chain fatty acid that is an important mediator of cellular metabolism. It is also a by-product of human gut enterobacteria and a common food preservative. A recent study found that rats administered with PPA showed autistic-like behaviors like restricted interest, impaired social behavior, and impaired reversal in a T-maze task. This study aimed to identify a link between PPA and autism phenotypes facilitated by signaling mechanisms in hippocampal neurons. Findings indicated autism-like pathogenesis associated with reduced dendritic spines in PPA-treated hippocampal neurons. To uncover the mechanisms underlying this loss, we evaluated autophagic flux, a functional readout of autophagy, using relevant biomedical markers. Results indicated that autophagic flux is impaired in PPA-treated hippocampal neurons. At a molecular level, the mitogen-activated protein kinase (MAPK)/extracellular signal-regulated kinase (ERK) pathway was activated and autophagic activity was impaired. We also observed that a MAPK inhibitor rescued dendritic spine loss in PPA-treated hippocampal neurons. Taken together, these results suggest a previously unknown link between PPA and autophagy in spine formation regulation in hippocampal neurons via MAPK/ERK signaling. Our results indicate that MAPK/ERK signaling participates in autism pathogenesis by autophagy disruption affecting dendritic spine density. This study may help to elucidate other mechanisms underlying autism and provide a potential strategy for treating ASD-associated pathology.

## Introduction

The human microbiome represents a diverse ecosystem of microbes. Reports on its influences on the immune system [1], metabolic processes [2], gene expression [3, 4], and nervous system [5] have led to increased recognition of the importance of the gut microbiome in human health and disease. The relationship between the gut microbiome and nervous system as a significant component of the gut-brain axis has also attracted increasing interest in recent years [6]. The gut microbiome possesses significant metabolic capacity, and certain microbe-derived metabolites are released into circulation and can cross the blood–brain barrier [7, 8]. Fermentation of dietary fiber by the colonic microbiome is the primary source of short-chain fatty acids (SCFAs), such as acetic acid, propionic acid (PPA), and butyric acid [9, 10]. PPA is used as a preservative in many processed foods, such as milk and cheese, and naturally occurs in yogurt [11, 12]. At appropriate levels, PPA improves immune function, facilitates cell signaling, and reduces food intake [9]. However, recent studies have reported that PPA is also associated with negative effects on health and behavior. Rats treated with PPA demonstrate impaired social behavior and inflammatory responses in the brain [13, 14]. High levels of PPA, but not other SCFAs, have been reported in the stools of autistic spectrum disorder (ASD) individuals [15]. Studies have demonstrated that intraventricular infusions of PPA caused abnormal behavioral patterns, such as abnormal social interaction and anxiety-like behavior in rats similar to those seen in humans with ASD [14, 16]. Although there is growing evidence that PPA can affect behavioral development, the direct effects of PPA on neuronal cells remain poorly understood.

It was previously published that SCFAs may modulate the levels of neurotransmitters [17]. For instance, neurogenesis-relevant signaling molecules such as brain-derived neurotrophic factor (BDNF) are shown to be modulated by change actions of SCFAs in the brain [18–20]. These data could reveal some clues about how SCFAs could regulate neuronal development. In neuronal circuits, dendrites from an individual neuron may have a thousand or more spines that participate in the establishment of excitatory synapses. Spines and synapses are produced in excess numbers during development that are later pruned via activity-dependent stabilization or elimination [21]. Dendritic spines are highly dynamic and undergo constant turnover and morphological plasticity depending on developmental stage and activity, and defective synapse formation or development has been implicated in many neurological diseases. However, whether PPA regulates the development of spine has not been experimentally tested. Changes in the number, formation, or elimination of dendritic spines depend on different molecules including actin [22], the cytoskeleton [23], cell surface receptors [24], and signaling pathways such as Ras and Rap kinase [25]. Moreover, activation of autophagy has also been reported to be related to spine morphology [26].

Macroautophagy is a cellular pathway, wherein damaged organelles are degraded, particularly those produced during oxidative stress, and metabolic precursors are regenerated [27]. In the initial step of autophagy, organelles are engulfed inside a double-membraned vesicle called an autophagosome. The autophagosome then fuses with an autolysosome to degrade organelles with lysosomal enzymes. Components are thereby recycled to produce energy and maintain synthesis [28]. Disruption of autophagy has been associated with several cellular pathologies, including tumors and neurological diseases [29, 30]. Impaired autophagy significantly reduces the size of synapses and number of boutons in *Drosophila* [31]. A previous study found that deletion of the vital autophagy gene *Atg7* resulted in increased immature dendritic filopodia and defects in synaptic refinement [32]. Evidence points to a relationship between autophagy and dendritic spine defects, but the mechanistic basis for these defects remains elusive.

Autophagy is regulated by a range of signaling pathways such as mammalian target of rapamycin (mTOR), ERK, and protein kinase B (AKT) [33]. ERK signaling is central to the MAPK pathway that regulates many cellular processes such as proliferation, differentiation, development, learning, and apoptosis [34]. The MAPK/ERK pathway is also a key regulator of autophagy, and starvation, a stimulator of autophagy, transiently activates MAPK/ERK to stimulate the maturation of the autophagosome [35]. Inhibition of MAPK/ERK activation by MAPK inhibitor pretreatment abolishes starvation-induced autophagy [36]. While it is difficult to draw a firm conclusion about the relationship of ERK to autophagy, it is clear that the MAPK/ERK pathway is an important factor therein.

We examined autophagic activity and the MAPK/ERK pathway to characterize the biological effects of PPA on hippocampal neurons. Results suggest that spine defects are associated with autophagy impairment and activation of the MAPK/ERK signaling pathway.

## Methods

### Primary culture

Primary cultures of rat hippocampal neurons were prepared from the brains of day 18 embryonic rats. Briefly, the hippocampus was dissected in free HBSS and incubated with a 0.125% trypsin solution for 15 min at 37 °C. The resulting cell suspensions were diluted in neurobasal medium (#21103–049, Gibco), supplemented with SM1 components (#05711, Stemcell), and plated onto 100 μg/mL poly-D-lysine (#P0899, Sigma-Aldrich) and 2 μg/mL laminin (#11–243–217-001, Roche)-coated plates or coverslips.

### Pharmacological treatment of hippocampal neuron

Propionic acid (#402907) and bafilomycin A1 (B1793) were purchased from Sigma-Aldrich, USA, and PD98059 (#513000) was purchased from Calbiochem. PPA was dissolved in phosphate-buffered saline (PBS) for treatment (100 μmole/mL). Bafilomycin A1 (2 nmole/mL) and PD98059 (10 μmole/mL) were dissolved in dimethyl sulfoxide and stored in aliquots at − 20 °C until the experiments. Vehicle (PBS), bafilomycin A1 (2 nmole/mL), and PD98059 (10 μmole/mL) were simultaneously treated with PPA (100 μmole/mL). PPA treatment was denoted as DIV 18, and cells were harvested on DIV 21.

### Western blotting analysis

Cultured neurons were harvested by scraping in ice-cold radio-immunoprecipitation assay buffer (#89900, Thermo Scientific) solution containing a protease inhibitor (A32963, Thermo Scientific) and phosphatase cocktail inhibitors (#5970, Cell Signaling) to avoid phosphorylation and degradation of proteins. After incubation, all lysates were centrifuged at 15,000 *g* at 4 °C for 30 min. The supernatant was then evaluated for total protein concentration using a BCA protein assay kit (#23225, ThermoFisher). Equal amounts of protein samples were incubated with 5X SDS sample loading buffer (CBSS-9005, CHEM-BIO) at 95 °C for 5 min. The samples (10 μg) were subjected to SDS-polyacrylamide gel electrophoresis on precast, 4–15% gradient mini-gels (#456–1085, Bio-rad). Following transfer to PVDF membranes (#1620177, Bio-rad), the membranes were blocked in Tris-buffered saline (#CBTB-9110, CHEM-BIO) containing 3% BSA (#9048-4-8, GENEray Biotechnology) and 0.1% Tween 20 (H5152, Promega) for 1 h. Membranes were then washed with TBST and incubated overnight at 4 °C with primary antibodies against phosphorylated ERK1/2 (#4370, Cell Signaling), phosphorylated AKT (#4060, Cell Signaling), LC3A/B (#12741, Cell Signaling), p62 (ab56416, Abcam), and beclin-1 (#3495, Cell Signaling). Membranes were then probed with horse radish peroxidase-conjugated secondary antibody (1:5000) for 1 h and developed using an enhanced chemiluminescence immunoblot detection system (Fusion FX7, VILBER). Immunoblots for phosphorylated ERK1/2 and phosphorylated AKT were subsequently stripped and re-probed with anti-ERK1/2 (#4692, Cell Signaling) and anti-AKT (#4691, Cell Signaling) antibodies. Immunoblots were analyzed by densitometry using ImageJ software (National Institutes of Health). Only film exposures that were in the linear range of the ECL reaction (#32106, Thermo Scientific) were used for quantification analysis.

### Immunofluorescence staining

Cultured neurons were fixed with 1% paraformaldehyde in PBS containing 4% sucrose for 5 min at room temperature. Without washing, neurons were then permeabilized and blocked simultaneously in 100% methanol for 7 min at − 20 °C. Primary antibodies against LC3A/B (#12741, Cell Signaling), p62 (ab56416, Abcam), lysosomal-associated membrane protein (LAMP1) (H4A3, Santa Cruz), and ubiquitin (P4D1, Santa Cruz) were added to blocking solution containing 0.1% gelatin, 0.3% Triton X-100, 16 mM sodium phosphate, and 450 mM NaCl and incubated overnight at 4 °C. After washing with PBS, coverslips were incubated with AlexaFluor488 (#4412, Cell Signaling) or AlexaFluor594 (#8890, Cell Signaling)-conjugated secondary antibodies for 1 h at room temperature and then washed extensively with PBS and distilled water. Subsequently, coverslips were mounted with mounting medium (H-1000, Vector Laboratories). The samples were imaged with a confocal laser-scanning microscope (Nikon, Japan) using a 60X oil lens and 488 nm and 594 nm emission lasers.

### Confocal microscopy for dendritic spines

Cells were maintained in an incubator with 5% CO_2_ at 37 °C. The neurons were transfected with 2 μg of GFP construct with 2 μL of lipofectamine 2000 (Invitrogen). Then, cells were stained by immunofluorescence labeling with anti-GFP antibody (A11122, Invitrogen). The samples were imaged with a confocal laser-scanning microscope (Nikon, Japan) using a 60X oil lens and 488 nm laser. Our data indicate that all spine categories were represented in pyramidal neurons. Dendritic spine densities were assessed by analyzing high-resolution digital images with ImageJ software (National Institutes of Health). Dendritic spines were counted manually using the point picker function in ImageJ particle analysis. The dendritic field was estimated to be 10 μm in length.

### Detection of autophagic flux

The formation of autophagosomes and autolysosomes in hippocampal neurons of control and PPA-treated cells was detected using a Premo™ Autophagy Tandem Sensor RFP-GFP-LC3B Kit (P36239, Invitrogen), according to the manufacturer’s instructions. The RFP-GFP-LC3B sensor enables the detection of LC3B-positive, neutral pH autophagosomes in green fluorescence (GFP), and LC3B-positive acidic pH autolysosome in red fluorescence (RFP). Cells were grown on coverslips and incubated with 10 μL of BacMam reagents containing RFP-GFP-LC3B for 16 h. Cells were then washed in PBS three times. Coverslips were mounted with mounting medium (H-1000, Vector Laboratories), and fluorescent images were taken using confocal microscopy (Nikon, Japan). LC3B-positive autophagosomes (GFP and YFP) and LC3B-positive autolysosomes (RFP only) were analyzed and quantified using ImageJ software (National Institutes of Health).

### Transmission electron microscopy for autophagy

Neuron cells were treated with 100 μM of PPA for 3 days and then fixed at 4 °C in 2.5% glutaraldehyde (#16210, EMS) and 2% paraformaldehyde (#19210, EMS); cells were then post-fixed with 2% osmium tetroxide (#19150, Sigma-Aldrich) for 30 min at 4 °C. Then, the cells were stained *en bloc* with 0.1 mg TCH (T1136, TCI) in 10 mL distilled water and 1% uranyl acetate (#22400, EMS) and dehydrated via a graded ethanol series. Samples were then embedded with an EMBed-812 embedding kit (#14120, EMS). Embedded samples were sectioned (60 nm) with an ultra-microtome (Leica, Germany), and the sections were then viewed on a Tecnai 20 transmission electron microscope (TEM) (ThermoFisher, USA) at 120 kV. Numbers of autophagosomes and autolysosomes per 100 μm^2^ were measured, as well as the size of the ultrastructure, using ImageJ software (National Institutes of Health).

### Statistical analysis

All data are represented as mean ± standard error of the mean (SEM) of at least three individual experiments. Statistical analysis was performed using one-way analysis of variance, and statistical significance (*p*-value) was calculated with GraphPad Prism 5 (GraphPad Software). Results at the 95% confidence level were considered significant.

## Results

### PPA induces the dendritic spine defects in hippocampal neurons

**Fig. 1:**
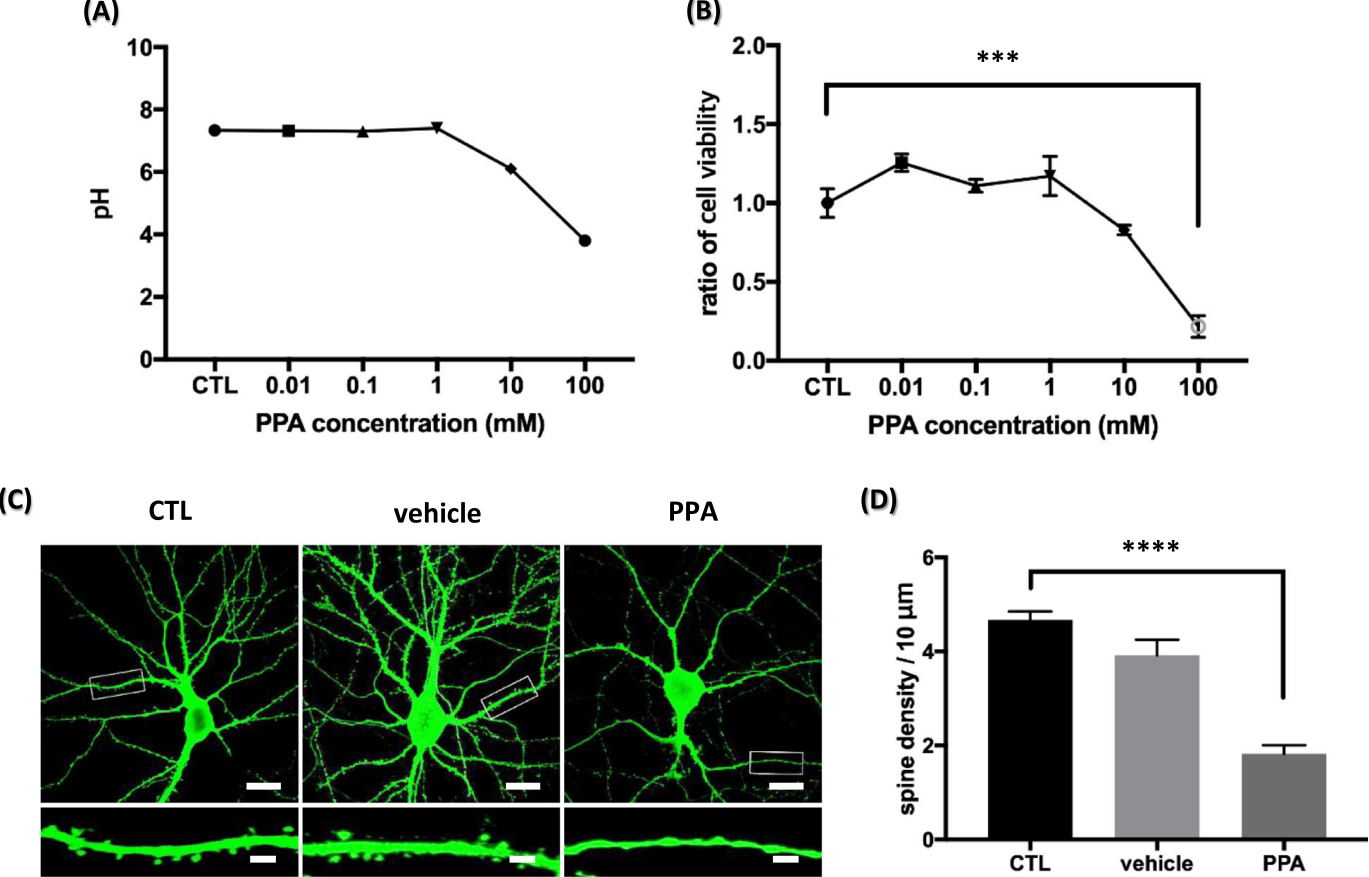
PPA decreases density of dendritic spines in hippocampal neurons (**a**) pH. **b** Cell viability. pH was measured in cultured media. Hippocampal neurons were incubated with PPA in concentrations ranging from 0 to 100 mM for 72 h. Viability was measured by CCK-8 assay. Results are expressed as percentage of viable cells and represent mean ± SEM of three samples (*n* = 3). **c** Top shows representative pyramidal neurons transfected with PLV-GFP and treated with vehicle (PBS) and PPA (100 μM) for 72 h. Bottom shows high magnification of representative secondary dendrites. Scale bar of top panel is 20 μm; scale bar of bottom panel is 1 μm. **d** Bar graph showing mean ± SEM for dendritic spine numbers of the representative groups (*n* = 50). ***p < 0.0005, ****p < 0.0001

We evaluated the effect of PPA on hippocampal neurons using pH and cell viability assays. The viability of hippocampal neurons treated with PPA was assessed with a CCK-8 assay. Hippocampal neurons were treated with PPA at concentrations of 0, 0.01, 0.1, 1, 10, and 100 mM. For hippocampal neurons treated with PPA at different doses, culture medium pH was decreased from the 10 mM PPA-treated group (Fig. 1a). Viability was decreased only in the 100 mM PPA-treated group (Fig. 1b). These results indicated that a 100 μM PPA concentration is not toxic to hippocampal neurons, and this was used in PPA treatments to investigate PPA’s effect on cellular organelle structure in hippocampal neurons.

We assessed the effect of PPA on hippocampal neuron morphology by investigating whether PPA affects dendritic spine formation. Hippocampal neurons were transfected with GFP plasmid DNA at DIV 17, and neuronal morphology was observed at DIV 21, a stage wherein highly dynamic dendritic spines gradually increase and become stable [37]. At DIV 21, neurons were fixed, stained, and imaged with confocal microscopy. Neurons were identified by green fluorescence, which diffusely filled the transfected hippocampal neurons and highlighted cellular morphology. We then analyzed these transfected pyramidal neurons by measuring secondary dendritic spine density. PPA-treated cells had visibly decreased dendritic spine density at both low and high magnifications of representative neurons and dendrites as shown by microscopic analysis (Fig. 1c). After treatment with PPA, the number of dendritic spines was 1.828 ± 0.1794, whereas that of untreated neurons was 4.671 ± 0.1818 in 10 μm. Quantitative analyses showed 39.13% lesser dendritic spine density in treated neurons than in controls (Fig. 1d). These findings indicate that PPA induces dendritic spine loss in hippocampal neurons.

### PPA upregulates biochemical markers of autophagy initiation in hippocampal neurons

**Fig. 2:**
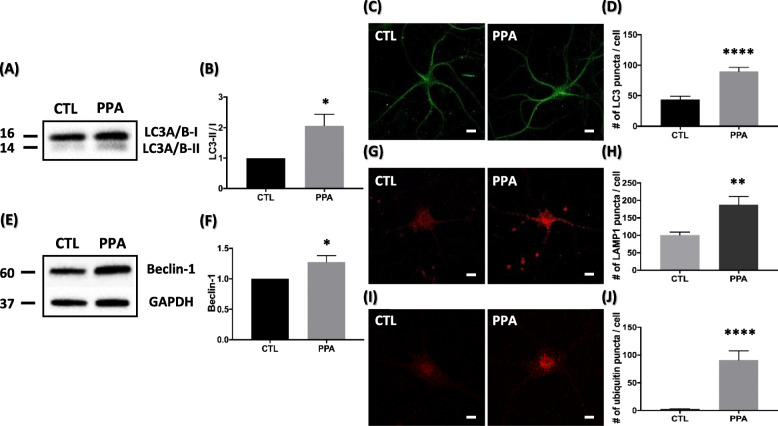
PPA upregulates biochemical markers of autophagy in hippocampal neurons (**a**) Representative western blot of LC3 after PPA treatment. **b** Quantification of LC3-II levels with respect to LC3-I levels (*n* = 4). **c**) Representative immunofluorescence images showing LC3 staining in hippocampal neurons and PPA-treated cells. **d** Quantification of number of LC3 puncta from images in (C) (*n* = 10). **e** Representative Western blot showing increased expression of beclin-1 for 72 h after PPA treatment. **f** Quantification of beclin-1 levels with respect to GAPDH levels (n = 4). **g** Representative immunofluorescence images showing LAMP1 staining in hippocampal neurons and PPA-treated cells. **h** Quantification of the number of LAMP1 puncta from images in (G) (*n* = 12). **i** Representative Immunofluorescence images showing poly-ubiquitin staining in hippocampal neurons and PPA-treated cells. **j** Quantification of the number of poly-ubiquitin puncta from the images in (I) (n = 10). Bar graph showing mean ± SEM of the representative groups. Scale bar is 10 μm. *p < 0.05, **p < 0.01, ****p < 0.0001

We addressed the impact of PPA on induction of autophagy by assessing several autophagy markers, such as intracellular microtubule-associated protein 1 light chain 3 (LC3), beclin-1, and LAMP1. As lipidation of LC3 and its association with autophagosome membranes is an indicator of autophagy [38], we detected LC3 by immunoblot and fluorescence microscopy. LC3-I was modified to the PE-conjugated form, LC3-II, because LC3-II is associated with mature autophagosomes upon autophagy induction. LC3 conversion from I to II is a measurement of autophagic activity [39]. We detected a significant increase in LC3-II/LC3-I ratio in PPA-treated cells compared with control, as shown by western blot (Fig. 2a, b). The number of LC3 puncta in PPA-treated cells was visibly increased as shown by immunofluorescence (Fig. 2c). After treatment with PPA, the number of puncta was 89.6 ± 6.765, whereas that of untreated neurons was 43.42 ± 5.549 (Fig. 2d). We then measured protein levels of beclin-1 by western blot (Fig. 2e). The beclin-1 protein is a tumor suppressor and a central regulator of autophagy critical to the nucleation phase of autophagy [40]. Wong and colleagues used beclin-1 antibody to visualize the formation of the autophagosome complex of protein aggregates to elucidate the autophagosome formation process [41]. After PPA treatment, beclin-1 was elevated by 27% compared with control neurons (Fig. 2f).

We next assessed the impact of PPA on protein aggregation-lysosome state levels of autophagic markers. PPA markedly increased the number of LAMP-1 and poly-ubiquitin puncta (Fig. 2g, i). After treatment with PPA, the number of LAMP-1 puncta was 188 ± 22.65, whereas that of the untreated neurons was 100.8 ± 8.645 (Fig. 2h). After treatment, the number of poly-ubiquitin puncta was 90.8 ± 16.91, whereas that of untreated neurons was 2.667 ± 0.9482 (Fig. 2j). These findings strongly suggest PPA as an inducer of autophagy throughout the process, from protein aggregation to autophagosome initiation.

### Autophagic degradation is impaired by PPA

**Fig. 3:**
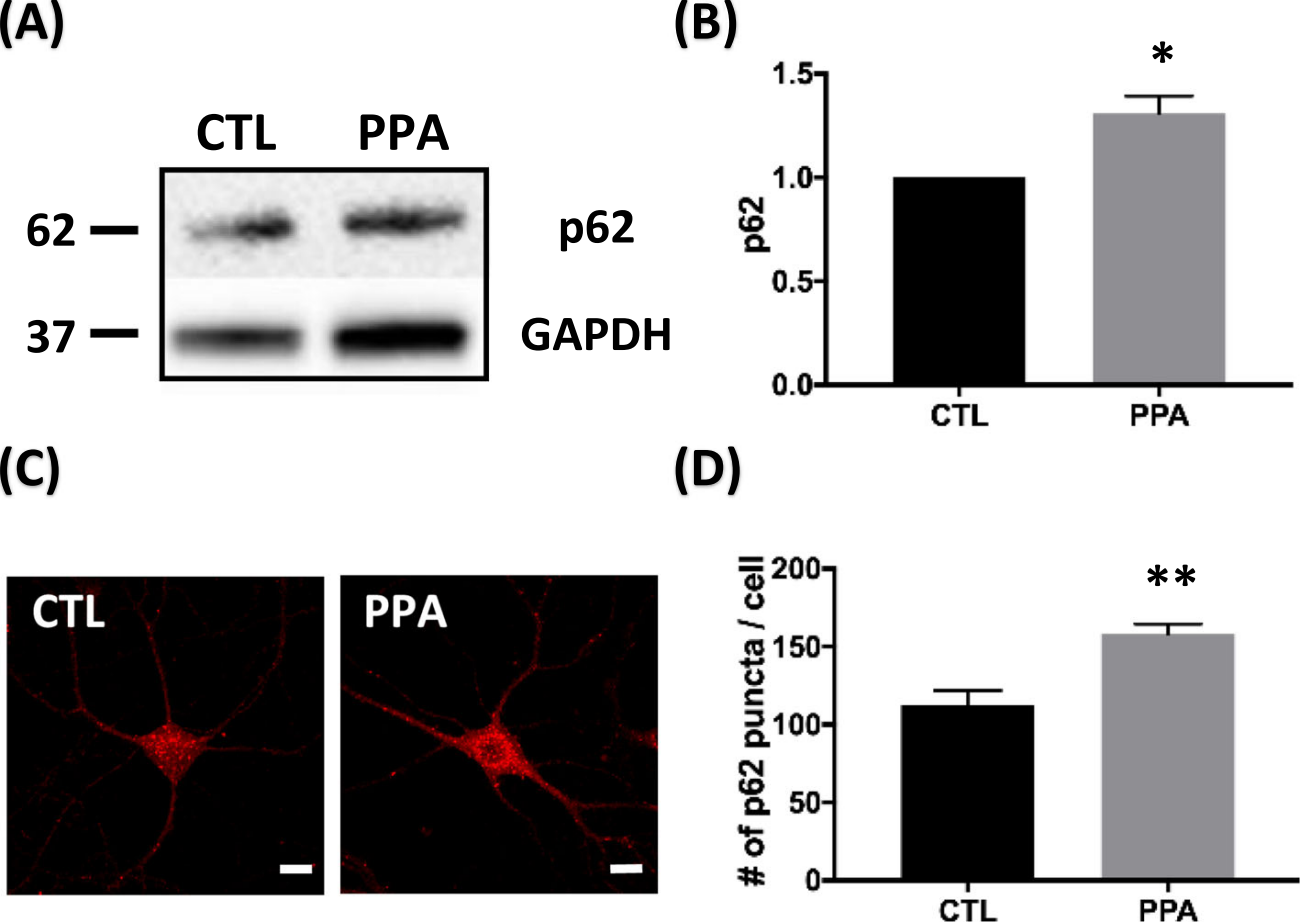
Autophagic degradation impaired by PPA (**a**) Representative western blot of p62 after PPA treatment. **b** Quantification of p62 levels with respect to GAPDH (n = 4). **c** Representative immunofluorescence images showing p62 staining in hippocampal neurons and PPA-treated cells. **d** Quantification of number of p62 puncta from images in (C) (*n* = 8). Bar graph showing mean ± SEM of representative groups. Scale bar is 10 μm. *p < 0.05, **p < 0.01

The interaction between LC3 and p62 was assessed by measuring protein levels of p62, a selective substrate of autophagy; p62 binds directly to LC3 at a short LC3 interaction region [42], and the induction of autophagic activity leads to p62 expression decline [43]. However, our immunoblot (Fig. 3a) and immunofluorescence (Fig. 3c) results showed a significant increase in p62 in PPA-treated cells compared with control. Specific immunoblot results showed a significant increase (30.8%) in p62 in PPA-treated cells over controls (Fig. 3b). After treatment with PPA, the number of p62 puncta was 158 ± 6.525, whereas that of untreated neurons was 112.3 ± 9.66 (Fig. 3d). PPA treatment was associated with elevated levels of p62, a protein degraded by autophagy that accumulates when autophagy is impaired. This finding indicated that the increase in LC3-II levels was not caused by enhanced formation but rather impaired clearance of the autophagosome. An increased LC3-II/LC3-I ratio (Fig. 2b, d) and an accumulation of p62 protein (Fig. 3b, d) were indicative of impaired autophagy in PPA-treated cells.

**Fig. 4:**
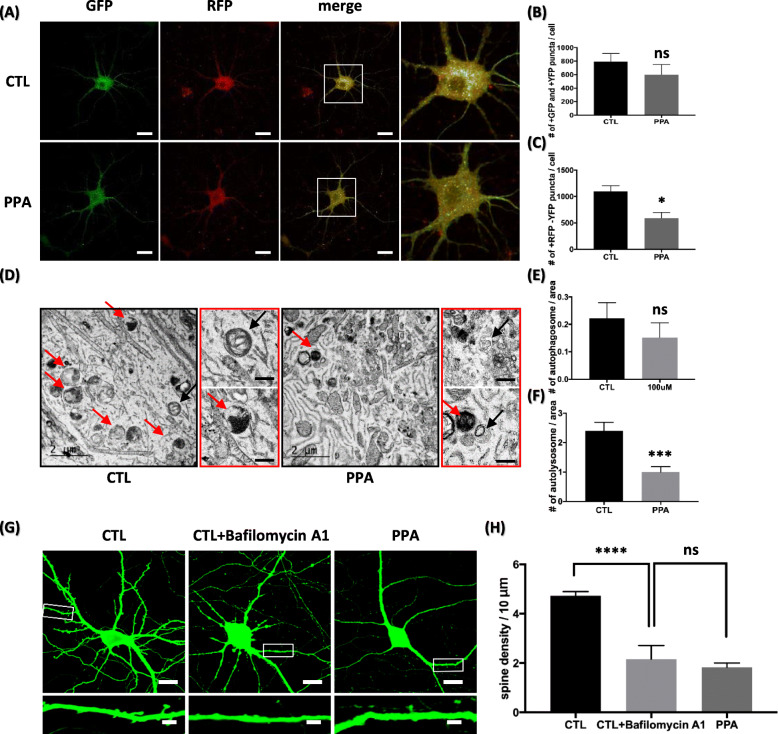
PPA dysregulates fusion from autophagosome to autolysosome (**a**) Representative fluorescent images after transfection with RFP-GFP-LC3B in control hippocampal neurons and PPA-treated cells. Scale bar is 20 μm. **b, c** Quantification of number of GFP and YFP puncta (autophagosome) and RFP only puncta (autolysosome) from images in (A) (n = 5). **d** Black box shows representative TEM images in control hippocampal neurons and PPA-treated cells. Red box shows representative TEM high magnification images of autolysosome and autophagosome (black arrow indicates autophagosome and red arrow indicates autolysosome). Black scale bar is 500 μm. **e**, **f** Quantification of autophagosome and autolysosome number per area (*n* = 35). Bar graph showing mean ± SEM of the representative groups. **g** Top shows representative pyramidal neurons transfected with PLV-GFP and treated with bafilomycin A1 (2 nM) and PPA (100 μM) for 72 h. Bottom shows high magnification of representative secondary dendrites. Scale bar of top panel is 20 μm; scale bar of bottom panel is 1 μm. **h** Bar graph showing mean ± SEM for dendritic spine numbers of the representative groups (n = 50). *p < 0.05, ***p < 0.0005, ****p < 0.0001

To understand the autophagy defect better, we investigated by transfecting hippocampal neurons with a marker gene, tandem fluorescent-tagged LC3 (GFP-RFP-LC3) in a baculoviral vector to monitor LC3 translocation. Results in Fig. 4a show that there is no significant change in autophagosome (GFP+ and YFP+ puncta) (Fig. 4b). After treatment with PPA, the number of RFP+ only puncta was 592 ± 104.4, whereas in the absence of PPA treatment, it was 1097 ± 109.4. A quantification analysis revealed a 50.2% decrease in red fluorescence expression (Fig. 4c), suggesting that PPA dysregulates the fusion of autophagosome to autolysosome.

We then further assessed fusion inhibition by analyzing the number of autolysosomes using TEM in control and PPA-treated cells (Fig. 4d). The number of autolysosomes was significantly decreased in PPA-treated cells, indicating inefficient fusion associated with PPA (Fig. 4f). These results strongly suggest that PPA dysregulates autolysosome fusion.

Moreover, to investigate whether autophagy defects by PPA are critical for spine loss, we investigated the effect of bafilomycin A1 in hippocampal neurons. Bafilomycin A1 is a well-known drug that disrupts autophagic flux by preventing cargo degradation [44]. Bafilomycin A1-treated cells visibly decreased dendritic spine density at both low and high magnifications of representative neurons and dendrites, as shown by microscopic analysis (Fig. 4g). After treatment with bafilomycin A1, the number of dendritic spines was 2.161 ± 0.5523, whereas that of untreated neurons was 4.726 ± 0.1715 in 10 μm. Quantitative analyses showed 45.72% lesser dendritic spine density in treated neurons than in controls (Fig. 4h). The reduction of spine density in bafilomycin A1-treated hippocampal neurons is similar compared with PPA-treated cells. These results show that PPA induces dendritic spine loss in hippocampal neurons by dysregulating autolysosome fusion, similar to bafilomycin A1.

### ERK signaling pathway is involved in PPA-induced autophagy defects in hippocampal neurons

**Fig. 5:**
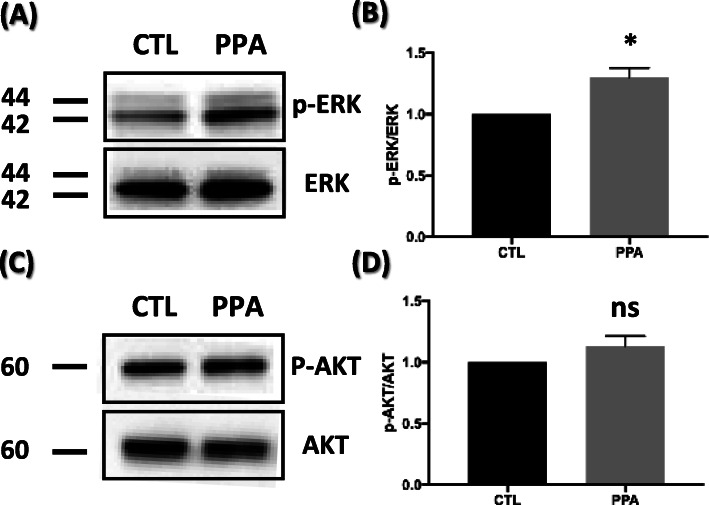
PPA activates MAPK/ERK pathway associated with autophagy impairment **a** Representative western blot of p-ERK and ERK after PPA treatment. **b** Quantification of p-ERK levels compared to ERK levels (n = 3). **c** Representative western blot of p-AKT and AKT after PPA treatment. **d** Quantification of p-AKT levels compared to AKT (n = 3). Bar graph showing mean ± SEM of the representative groups. *p < 0.05

MAPK/ERK signaling regulates the expression of autophagic and lysosomal genes and stimulates autophagy by interacting with LC3. We investigated whether PPA exposure changes ERK1/2 signaling in hippocampal neurons by detecting phospho-ERK1/2 and total-ERK1/2 using immunoblot assays (Fig. 5a). As shown in Fig. 5b, we observed a significant increase (30.2%) in the p-ERK/ERK ratio in PPA-treated cells compared with controls (Fig. 5b). We then assessed whether PPA changes AKT signaling in hippocampal neurons by detecting phospho-AKT and total AKT using immunoblot assays (Fig. 5c). Compared with the level of p-AKT/AKT, no significant change in p-AKT/AKT ratio in PPA-treated hippocampal neurons was observed (Fig. 5d). These results suggest that the MAPK/ERK pathway is more associated with autophagy impairment than the AKT pathway in this study.

### Inhibition of MAPK/ERK signaling rescues PPA phenotypes in vitro

**Fig. 6:**
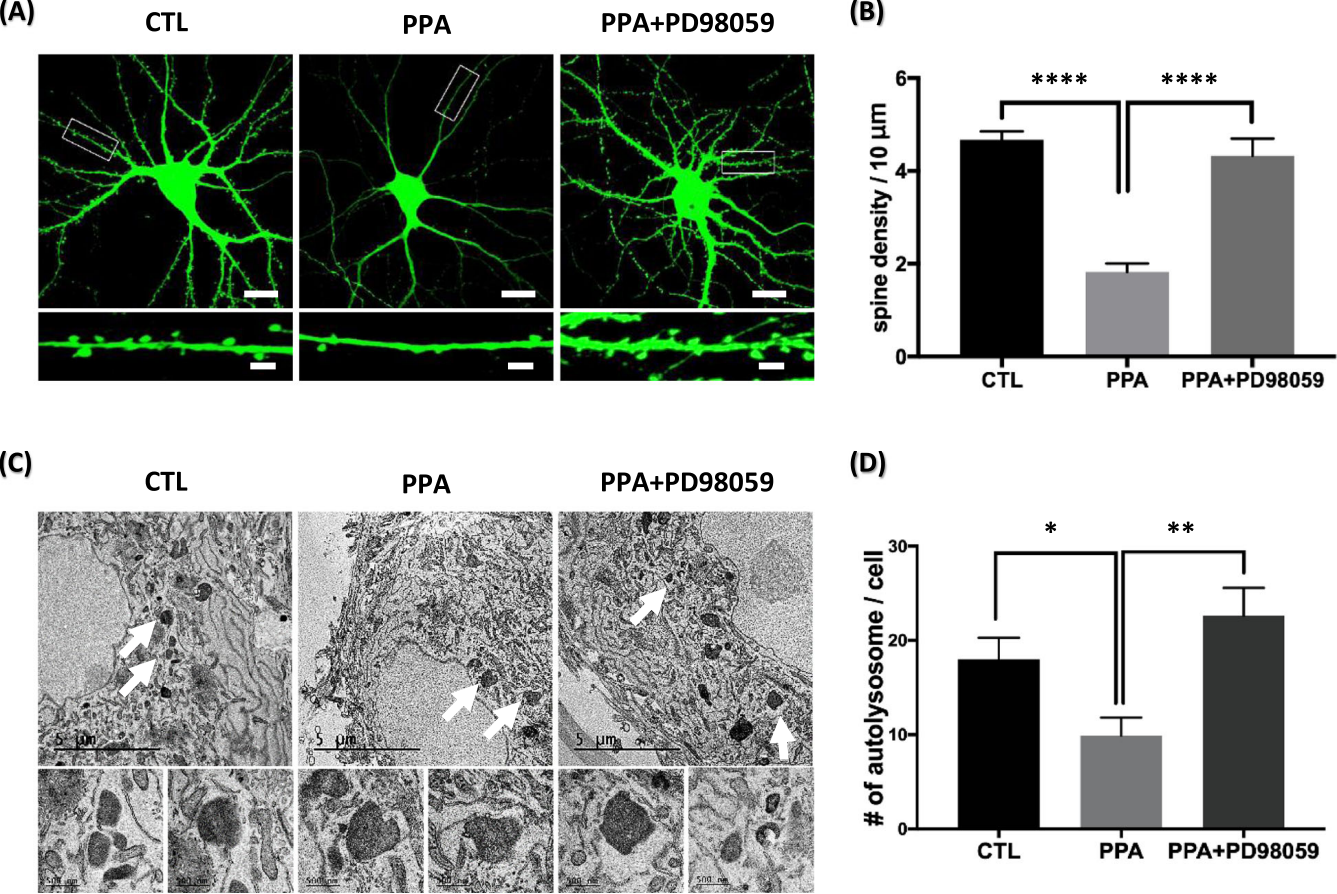
PD98059 rescues spine density and autophagy disruption in PPA-treated hippocampal neurons **a** Top shows representative pyramidal neurons transfected with PLV-GFP and treated with PPA (100 μM) and PD98059 (10 μM) for 72 h. Bottom shows high magnification of representative secondary dendrites. Scale bar of top panel is 20 μm; scale bar of bottom panel is 1 μm. **b** Bar graph showing mean ± SEM for dendritic spine numbers of the representative groups (n = 50). **c** Top shows representative TEM images in PPA (100 μM) and PD98059 (10 μM) for 72 h (white arrow is autolysosome). Bottom shows high magnification of representative images of autolysosome. **d** Quantification of autolysosome number per cell (n = 10). Bar graph showing mean ± SEM of the representative groups. *p < 0.05, **p < 0.01, ****p < 0.0001

Finally, we analyzed the impact of PD98059, a MAPK inhibitor, on dendritic spine loss and autophagic flux in hippocampal neurons. PD98059 treatment rescued dendritic spine density in PPA-treated neurons, an effect that was visible at both low and high magnification of representative neurons and dendrites (Fig. 6a). Quantitative analyses showed a 42.2% rescue of dendritic spine density in PD98059-treated neurons over PPA-treated-only neurons (Fig. 6b). In addition, to investigate whether PPA-induced MAPK/ERK activation is involved in autophagy regulation, we observed fusion activity by analyzing the number of autolysosomes using TEM. PD98059 treatment rescued the number of autolysosome in PPA-treated neurons (Fig. 6c). The number of autolysosomes was significantly increased (40.9%) in PPA and PD98059-treated neurons than in only PPA-treated neurons (Fig. 6d). Taken together, our data showed that inhibition of MAPK/ERK signaling rescued dendritic spine loss and autophagic disruption by PPA.

## Discussion

Recent studies have demonstrated that autophagy is involved in synaptic remodeling in *Drosophila melanogaster* and mice [45]. Autophagy dysfunction is associated with reduced synaptic plasticity and dendritic spine deficiency [46]. Autophagic activity is required for BDNF-mediated synaptic plasticity in the hippocampus [47]. Auerbach and colleagues demonstrated that TSC1/2 deficiency associated with decreased autophagy leads to synaptic plasticity impairment in the hippocampus [48]. Another study showed that cortical neuron dendritic spine density was reduced in TSC1 knockout mice [49]. Goo et al. reported that lysosome inhibition alters lysosome trafficking in dendrites; furthermore, it decreases dendritic spine density [50]. In our study, PPA treatment of hippocampal neurons resulted in increased autophagy initiation but impaired autolysosome fusion. Electron and immunofluorescence microscopy analyses indicated that PPA-induced autophagosomes had not matured. They were double membrane-enclosed vesicles containing organelles, but the subsequent fusion with the outer membrane of lysosomes indicating autophagosome maturation, wherein the inner contents of autolysosomes are degraded for synthesis of new molecules and organelles, did not occur. Our findings suggest that PPA induces spine loss by disrupting the maturation of autophagosomes into functional autolysosomes. An increase in autophagosomes without a concomitant increase in autolysosomes provided valuable information for identifying signaling events. PPA was found to selectively disrupt autolysosomal maturation. This impairment of autophagy led to a decrease in dendritic spine density. We also showed the dendritic spine loss in bafilomycin A1-treated hippocampal neurons (Fig. 4g, h). Our results suggest that the mature of autolysosome in the spine helps to maintain cellular homeostasis and remodel synapse.

What is the delineation of signals that control autophagy? One report provides evidence that MAPK/ERK tightly regulates the maturation of autophagosomes [35]. Activation of the MAPK/ERK pathway was sufficient to arrest the autophagic maturation step with characteristic accumulation of autophagosomes and abolishment of autophagic degradation [36]. For instance, ERK cascade components colocalize with autophagosome, suggesting that aberrant autophagy is associated with ERK hyper-phosphorylation [51]. Consistent with this report, our results showed that the MAPK/ERK pathway is activated during autophagy maturation impairment in PPA-induced cells. We further found that the inhibition of MAPK/ERK signaling restored the autolysosome-represent autophagic flux (Fig. 6d). These findings indicate that MAPK/ERK activation is involved in autophagy defects. The AKT signaling pathway also regulates autophagy [33]; however, we observed that activation of this pathway did not significantly affect dendrites (Fig. 5d), implicating the MAPK/ERK pathway, but not the AKT pathway, in autophagy impairment. Spine density and disruption of autophagic flux were rescued when a MAPK inhibitor and PPA were co-treatment. It indicates that inhibition of the pathway specifically may affect maturation and not autophagy initiation.

A growing number of studies have implicated gut microbiota dysbiosis in behavioral and neurologic pathologies, such as in Alzheimer’s, Parkinson’s disease and ASD [17]. PPA administration has been proposed as a target for ASDs [52, 53]. Dendritic spines may serve as a common substrate for many neuropsychiatric disorders, particularly those that involve cognitive deficits, and dysregulation in spine morphology has been implicated in ASD [54]. There are two types of dendritic spine modifications in animal models of ASD-related genes. For example, *SHANK3*-mutated mice and *PTEN* transgenic mice models of ASD show a decrease in spine density. However, FMRP, MeCP2, neuroligin-3/4, and neurexin1 ASD models show an increase in spine density [55]. Our results illustrated a possible link between PPA and reduction of dendritic spine density in ASD animal model.

Taken together, we speculate that autophagy degradation disruption and MAPK/ERK signaling pathway activation induce dendritic spine defects and may affect synapse function and plasticity, leading to autistic-like phenotypes in PPA-treated hippocampal neurons. It will be of interest to determine if altered autophagy function and MAPK/ERK inhibition is a contributing factor to ASD. Our study represents the first empirical data supporting MAPK/ERK signaling pathway involvement in autophagic degradation disruption and the resulting pathologies associated with ASD.

## Data Availability

All data generated or analyzed during this study are included in this published article.

## Abbreviations

PPA: Propionic acid
MAPK: Mitogen-activated protein kinase
ERK: Extracellular signal-regulated kinase
mTOR: Mammalian target of rapamycin
AKT: Protein kinase B
ASD: Autism spectrum disorder
BDNF: Brain-derived neurotrophic factor
PBS: Phosphate-buffered saline
DIV: Days in vitro
GFP: Green fluorescent protein
YFP: Yellow fluorescent protein
RFP: Red fluorescent protein
LC3: Intracellular microtubule-associated protein 1 light chain 3
LAMP1: Lysosomal-associated membrane protein 1
TEM: Transmission electron microscope
SEM: Standard error of the mean
